# Blockchain-Authenticated Sharing of Genomic and Clinical Outcomes Data of Patients With Cancer: A Prospective Cohort Study

**DOI:** 10.2196/16810

**Published:** 2020-03-20

**Authors:** Benjamin Scott Glicksberg, Shohei Burns, Rob Currie, Ann Griffin, Zhen Jane Wang, David Haussler, Theodore Goldstein, Eric Collisson

**Affiliations:** 1 Bakar Computational Health Sciences Institute University of California San Francisco, CA United States; 2 Hasso Plattner Institute for Digital Health at Mount Sinai Icahn School of Medicine at Mount Sinai New York, NY United States; 3 Department of Genetics and Genomic Sciences Icahn School of Medicine at Mount Sinai New York, NY United States; 4 Division of Hematology and Oncology Department of Medicine University of California San Francisco, CA United States; 5 UC Santa Cruz Genomics Institute University of California, Santa Cruz Santa Cruz, CA United States; 6 Helen Diller Family Comprehensive Cancer Center University of California San Francisco, CA United States; 7 Department of Radiology and Biomedical Imaging University of California San Francisco, CA United States; 8 Howard Hughes Medical Institute Santa Cruz, CA United States

**Keywords:** data sharing, electronic health records, genomics, medicine, blockchain, neoplasms

## Abstract

**Background:**

Efficiently sharing health data produced during standard care could dramatically accelerate progress in cancer treatments, but various barriers make this difficult. Not sharing these data to ensure patient privacy is at the cost of little to no learning from real-world data produced during cancer care. Furthermore, recent research has demonstrated a willingness of patients with cancer to share their treatment experiences to fuel research, despite potential risks to privacy.

**Objective:**

The objective of this study was to design, pilot, and release a decentralized, scalable, efficient, economical, and secure strategy for the dissemination of deidentified clinical and genomic data with a focus on late-stage cancer.

**Methods:**

We created and piloted a blockchain-authenticated system to enable secure sharing of deidentified patient data derived from standard of care imaging, genomic testing, and electronic health records (EHRs), called the Cancer Gene Trust (CGT). We prospectively consented and collected data for a pilot cohort (N=18), which we uploaded to the CGT. EHR data were extracted from both a hospital cancer registry and a common data model (CDM) format to identify optimal data extraction and dissemination practices. Specifically, we scored and compared the level of completeness between two EHR data extraction formats against the gold standard source documentation for patients with available data (n=17).

**Results:**

Although the total completeness scores were greater for the registry reports than those for the CDM, this difference was not statistically significant. We did find that some specific data fields, such as histology site, were better captured using the registry reports, which can be used to improve the continually adapting CDM. In terms of the overall pilot study, we found that CGT enables rapid integration of real-world data of patients with cancer in a more clinically useful time frame. We also developed an open-source Web application to allow users to seamlessly search, browse, explore, and download CGT data.

**Conclusions:**

Our pilot demonstrates the willingness of patients with cancer to participate in data sharing and how blockchain-enabled structures can maintain relationships between individual data elements while preserving patient privacy, empowering findings by third-party researchers and clinicians. We demonstrate the feasibility of CGT as a framework to share health data trapped in silos to further cancer research. Further studies to optimize data representation, stream, and integrity are required.

## Introduction

Every patient with cancer has a unique disease composition and presentation that demands interrogation of complex imaging and genome characteristics [1,2] for personalized treatment recommendations. Currently, it is still standard to report outcomes of cancer as group averages from clinical trials treated with prospectively dictated regimens. Individual patient outcomes from real-world data could further advance personalized medicine by allowing dramatically more treatments and outcomes to be considered [3,4]. As such a health system can learn from its own data to improve its delivery of patient care [5-7]. Regulatory requirements and other restrictions prevent much patient-level data from being shared. Research progress suffers as a result. Precision medicine methodologies such as next-generation tumor DNA sequencing are now often performed in routine cancer care. Unfortunately, results are siloed in individual institutions, frustrating effective sharing or pooling of datasets [8]. Many patients with cancer, however, are willing to share their data and believe that the positive benefits outweigh the potential privacy risks: 93% of patients surveyed would be very or somewhat likely to share their data with university scientists [9].

Despite this need and patients’ willingness to share their data, robust deidentified data sharing methods are lacking. Innovative alternative strategies have been developed that aim to anonymize identifiable clinical data in a way that preserves inherent structure, such as using generative adversarial networks [10], but these have not as of yet been deployed for large-scale, multiomic discovery. One immediate challenge of creating an extensible and robust framework is identifying which data are necessary to share (and in what format), minimizing risk for patient reidentification while maximizing viable information that can lead to clinical insight. Conley et al [11] released a core set of clinical data elements that various stakeholders agreed on for cancer genomic repositories. The lack of a standard data sharing platform for clinical data arises from myriad causes, including but not limited to, incompatible data streams or formats, nonstandardized collection, conflicting business models, extraction and accessibility procedures, and privacy concerns. A centralized, curated platform operated by a single institution is not ideal due to concerns of data ownership, cost, and dissemination procedures. Trends in other fields have migrated from analyzing batched data quarterly, whether from customer Web clicks or manufacturing floor sensors, to real-time analyses. Learning cycles have been reduced from months to hours. Finally, centralized top-down data sharing efforts, although critical to research and scientific deductive understanding, have a fixed lifetime of the study, grant, or group interest.

Software standards based on health care data sharing and electronic commerce are converging to enable solutions to the compelling need to share patient health data for both care management and medical research. In 2013, the Global Alliance for Genomic Health [12] was established to enable a framework for secure, responsible, and effective clinical and genomic data sharing. In 2016, the US president unveiled the National Cancer Institute Cancer Moonshot effort to accelerate cancer research, including efforts focused on data sharing (the Public Access and Data Sharing Policy). Since then, significant progress has been made in mining and sharing medical data. The Food and Drug Administration announced a collaboration with Flatiron Health to utilize deidentified clinical data for the analysis and development of anticancer therapies outside of clinical trials in 2016. Recent studies have delivered on that promise: Agarwal et al [7] analyzed more than 7000 clinical and genomic records from the Flatiron Health network and Foundation Medicine to calculate the tumor mutation burden across cancer subtypes. Singal et al [13] demonstrated that data collected from routine clinical care of almost 30,000 patients with cancer can yield novel clinical insights, as evidenced in this case for non–small cell lung cancer.

A decentralized, scalable, efficient, economical, and secure strategy, such as blockchain technology, can fulfill requirements for effective clinical data sharing. Although not perfect in their scope [14], blockchain systems by design are secure and resistant to tampering and distributed with no single point of control or failure allowing transactions to be efficiently recorded and verified. Multiple publications have proposed the utility of blockchain technology for secure and scalable clinical data sharing [15-19], and many companies and organizations are applying blockchain platforms in health care [20]. Although the excitement surrounding the utilization of blockchain for distributing health care data is encouraging [21], many studies are private, theoretical (ie, accessing feasibility), or unsuccessful in scope. In a recent systematic review of 71 studies that discussed managing health care records via blockchain, only four actually were tested on live data [22].

Here, we develop a public demonstration of curated collection that focuses on capturing the data created over the normal course of clinical care as rapidly as possible. The Cancer Gene Trust (CGT) [23] democratizes data analysis, enabling more experts to participate and compare results, and accelerates the translation of genomic findings toward a clinically useful timescale. CGT is the first free, simple, rapid, global network to share deidentified cancer somatic mutations, radiographic and pathological images, and associated clinical data for prospectively consented patients. These data are rapidly deposited into a global off-blockchain distributed and decentralized repository. This framework not only allows for the rapid dissemination of high yield and important data but also openly details the rigorous process for deidentification, study design, and informed patient consent. From the findings of Mello et al [9], we hypothesized that most patients are willing to consent to their data being shared if it helps expand the corpus of medical knowledge. We aim to demonstrate the utility of CGT by releasing such data from a pilot study of 18 consented patients along with an open-source and freely available application for visualization and exploration.

## Methods

### Study Design and Recruitment

The University of California, San Francisco (UCSF) institutional review board (IRB) approved our pilot study to consent patients for distributing their deidentified information on CGT (see Multimedia Appendix 1 for study protocol). We approached and consented 18 patients under care at UCSF Medical Center to the *Sharing Clinical and Genomic Data in Cancer Research* clinical pilot protocol (IRB #16-20857).

### The Cancer Gene Trust Framework

CGT is a decentralized, distributed content addressable real-time database. A submission consists of a manifest containing fields and references to files by hash. Submissions may include deidentified clinical fields, a list of somatic mutations, gene expression, or any type of data relevant to a patient. Submissions are tracked per steward (ie, institution or organization) via a smart contract on the Ethereum [24] blockchain, which references the underlying data stored via hash in InterPlanetary File System (IPFS) [25]. IPFS is inherently decentralized and distributed. Any node may request data from any other node via the unique hash of the data and cache it locally. This affords organic replication of data as well as scalable access. An institution performing internal access and analysis of data may run their own IPFS server and thereby allow high-speed LAN access with only the initial request traversing the list of IPFS servers to find data matching the hash.

### Data Collection Procedures

**Figure 1:**
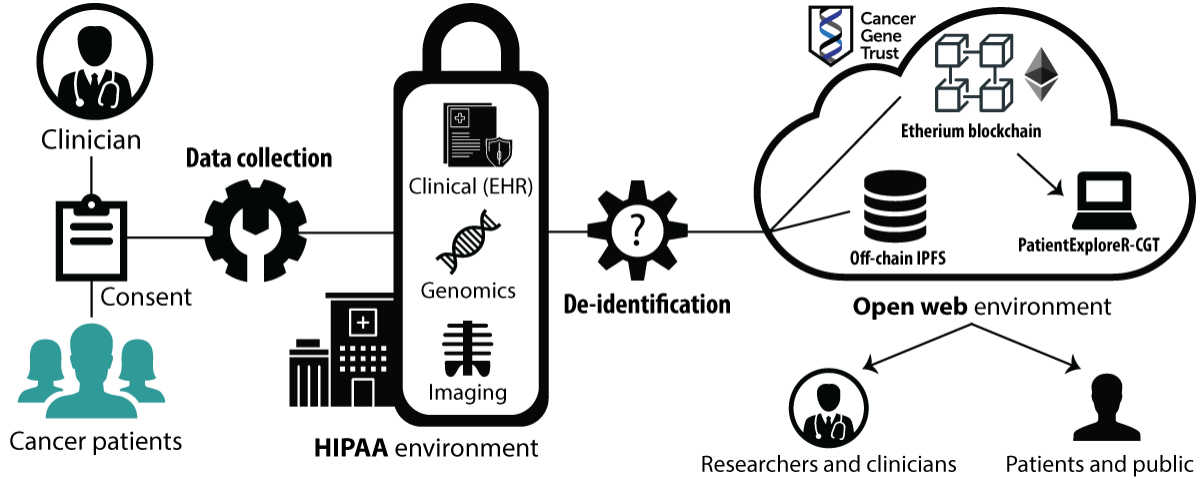
Workflow and pipeline for Cancer Gene Trust. EHR: electronic health record; HIPAA: Health Insurance Portability and Accountability Act.

We carefully navigated all institutional procedures to educate and consent our patients before obtaining, formatting, and distributing deidentified patient data from our cohort (Figure 1). We performed stringent and comprehensive privacy processes to be as confident as possible so that no identifying personal health information would be shared (see Data Deidentification section). For the 18 enrolled patients, we were given permission to obtain clinical documentation from their electronic health record (EHR), their somatic mutation information, as well as any scans taken [26]. All data, including genomic, imaging, and structured EHR data (eg, treatment information), for the first cohort of consented patients are available [23]. Patients are identified by a universally unique identifier (UUID-4). The only mapping to the actual patient is securely controlled by trusted stewards; in this case, UCSF. All source code and documentation for CGT are available [27].

#### Overall Workflow for Cancer Gene Trust Pipeline

Patients are consented to agree to release their deidentified clinical (Observational Medical Outcomes Partnership [OMOP]-formatted EHR data), genomics (somatic), and imaging data on the blockchain. Stewards representing the affiliated institution then upload the data to CGT. Researchers, clinicians, patients, and the public can then retrieve the data through the Web or interface, with the data dynamically available through the PatientExploreR-CGT app.

#### Genomic Data Collection

Somatic gene sequencing of tumor specimens was ordered by the supervising physician (EC) as standard of care using either a commercial (Foundation Medicine; FMI [28]) or in-house panel (UCSF 500) [29]; 13 patients were sequenced and analyzed by Foundation Medicine [30] and 4 patients by the UCSF 500 [31] genomic panel. In the case of Foundation Medicine, we received the patient’s report in XML format. In the case of the UCSF 500, we requested a deidentified variant call file from the UCSF genomic stewards.

#### Image Data Collection

For patients with available radiograph imaging, we obtained deidentified DICOM files from the UCSF’s Picture Archiving and Communication System medical imaging system conforming to Supplement 142: Clinical Trial De-identification Basic Profile, which removes any identifying protected health information (PHI) from the images as well as any accompanying metadata. Pathology slides were obtained for each patient who had associated pathology performed at UCSF. Deidentified computed tomography (CT) and positron-emission tomography-CT scans correlating to significant changes in tumor response were uploaded for 3 patients to the CGT. Scanned pathology slides clinically utilized for diagnostic purposes were uploaded for 2 of these patients. These deidentified imaging data can be viewed publicly in their entirety on the CGT and illustrate an example collection of raw (but deidentified), clinically relevant data for public research use. Phillips scanners were used to digitize the pathology slides, and a review of PHI was completed before uploading onto CGT.

#### Clinical Electronic Health Record Data Collection

A large aspect of this project was to evaluate the most suitable and robust source of clinical data to share on CGT. For this comparison, we compared UCSF Cancer registry data, collected to meet the specifications of the Surveillance, Epidemiology, and End Results (SEER) Program, with Observational Health Data Sciences and Informatics (OHDSI) OMOP common data model (CDM) extracted from the hospital EHR. The OMOP CDM is emerging as a standard in the field of EHR research because it is a common framework in terms of both table structure and underlying vocabulary [32] and has enabled powerful research and a venue for regulatory reporting [33].

SEER is a national registry for cancer reporting and provides specific guidelines for data collection from the EHR [34]. Before SEER submission, cancer registry data are submitted to the state registry and assessed for data quality and consolidation with other records for the same patient. Registry data are collected on every cancer case admitted to a UCSF hospital for either diagnosis and/or first course or subsequent cancer treatment per California state cancer reporting law. Certified Tumor Registrars abstract and code cancer information from the EHR in a format specified by the North American Association of Central Cancer Registries’ Data Standards [35]. The data collection and coding rules for data collection are specified by the SEER Program Manual and fully abstracted within 6 months of patients’ date of first contact with the hospital.

#### Registry Format

For the first 18 patients, clinical data were requested from the cancer registrar’s office for curated data for ultimate submission to SEER via the CNExT cancer registry software. For each patient, we received an Excel export from CNExT with curated clinical data fields (Table 1). We developed a client-side single-page Web application that read in this Excel file on the research coordinators computer, filtered PHI to ensure compliance with IRB regulatory guidelines, and generated a deidentified JSON file. The primary investigator and research coordinator personally reviewed each deidentified registry file for PHI before uploading onto CGT. Depending on the timing of the patient’s presentation to the hospital relative to genetic testing, the registry data collection could be in either an incomplete *suspense* state or a completed abstract. Minimum data collection in a suspense case comprises patient age, gender, date of first contact, primary site, and histology. Complete cases contained additional data items related to Basis of Diagnosis and Therapeutic Agent.

**Table 1:** Breakdown of data elements for registry/Observational Medical Outcomes Partnership.

Gold Standard EHR^a^	Registry field	OMOP table.column
Gender	Sex	person.gender_concept_id
Ethnicity	Spanish Origin	person.ethnicity_concept_id
Race	Race	person.race_concept_id
Date of Diagnosis	Date of Diagnosis^b^	condition_occurrence. condition_start_date
Basis of Diagnosis	Dx Confimation DX Staging/Proc Summ^b^	procedure_occurrence. procedure_occurrence_id
Cancer Site	Cancer Site ICD-0-3 SEER^c^ Site Group	condition_occurrence. condition_concept_id
Cancer Histology/Morphology	Cancer Histology (ICD-0-3)	condition_occurrence. condition_concept_id
Therapeutic Agent/Modality	Text/Code of Chemo At Hospital^b^	drug_exposure. drug_concept_id
Beginning and End Dates of Treatment	Chemo Start Date/Chemo End Date^b^	drug_exposure. drug_exposure_start_date/drug_exposure. drug_exposure_end_date

^a^EHR: electronic health record.

^b^Indicates that the field is listed but no or incomplete information was populated (ie, “suspense” registry cases).

^c^SEER: Surveillance, Epidemiology, and End Results.

#### Observational Medical Outcomes Partnership Format

Procuring clinical data from OMOP was a different process as it involved extraction of retrospective, routinely collected data from the EHR. The Enterprise Data Warehouse (EDW) team at UCSF is responsible for converting raw EPIC/Clarity data into the OMOP format and acted as an honest broker for this extraction process. First, we selected the tables and fields that corresponded to data elements we were consented to collect from our IRB, with buy-in from the EDW team (Table 1). No free-text fields were included. We then provided the medical record numbers (MRNs), and their corresponding CGT patient IDs, to the EDW team who then performed the deidentification process for 17 patients with available data, removing all PHI (see Multimedia Appendix 1 and below for more details). The EDW then extracted the data in the agreed-upon columns in 6 tables of interest, specifically: person, drug_exposure, condition_occurrence, procedure_occurrence, and measurement. We then performed a secondary check to verify all data were deidentified (see below), and then transformed the files (saved as TSV) into a single JSON file per patient.

### Clinical Data Scoring Methodology

We evaluated all patients’ registry and OMOP data for completeness based on a scoring rubric we designed (see Multimedia Appendix 1 for full details) relating to certain gold-standard metrics essential for clinical data sharing (Table 1), inspired by Conley et al [11]. Data from these gold-standard metrics were captured from the *true* data recorded in UCSF EPIC EHR system patient records. Next, reviewers evaluated how much of these data could be identified from registry and OMOP data sources. Of the 29 data elements recommended by Conley et al [11], we were able to capture 10 of these due to their ability to be obtained without curation from OMOP and registry clinical pipelines. Simply, these data were evaluated on a scale from 0 to 5 for registry and OMOP data, with 0 representing no presence of the data element in the corresponding modality and 5 representing complete representation (values in between correspond to 20% increments of how complete the representation is). As such, for the 10 data elements, the maximum score a patient can receive per data modality is 50.

### Statistical Analysis

To assess whether there was any significant difference between registry vs OMOP in terms of data quality capture, we performed a 2-sided Wilcoxon signed-rank test for all 17 patients who were scored according to the above methodology. We further assessed whether there was any difference at the field level, by performing the same assessment per data element (eg, Gender information). We hypothesized that although these two systems are different in terms of data collection methodologies, there should be no significant difference in total scores as both systems are organized to capture the same type of clinical data.

### Data Deidentification Procedures

We strived to conform to the most rigorous standards for proper deidentification of all data released as determined by Health Insurance Portability and Accountability Act (HIPAA) standards (see Multimedia Appendix 1 for further discussion and complete documentation of this process).

For the OMOP EHR data, all PHI was removed on receiving the data from the honest broker, the EDW. In these files, all dates were converted into age in days since birth. We performed a secondary check to manually verify that no PHI remained in the files. For genomic data, all germ-line mutations were removed, leaving only somatic variants. No further processing was required for the DICOM images that conform to Supplement 142. Pathology scans were exported into JPEG image files with no identifying metadata or information in the image. The single-page Web application generates a UUID for every patient. The institution and CGT steward maintain an appendix of CGT IDs and UCSF MRNs to preserve the possibility of reidentification between qualified clinicians for follow-up and further research [36].

### Data Export and Sharing

These deidentified files are uploaded to the off-blockchain store (IPFS) [25]. The off-blockchain store calculates a cryptographically strong hash (SHA-256) of the entire submission that is added to the stewards list of submissions, which is then updated in the off-blockchain store. This final step yields an updated top level cryptographically strong hash that uniquely defines the entire state of all submissions from the steward at that point in time. This final top-level hash is then submitted to the blockchain as provenance for the entire corpus of submissions from the institution. As the hash is only 256 bits in size, the cost to add to a blockchain is minimized with the bulk of the data stored uniquely in the off-blockchain store. Individual submission hashes as well as the overall steward hash may be concisely referenced toward reproducing any downstream analysis.

### Data Distribution and Access

Submissions including all data are immediately available from any IPFS server on the internet via the submission hash. IPFS is inherently decentralized and distributed. Any node may request data from any other node via the unique hash of the data and cache it locally. An IPFS server when queried for the data associated with a hash returns it if it has it locally stored, and if not asks all of the servers it is connected to for the data. In spirit, this is similar to the Transmission Control Protocol/Internet Protocol layer of the internet whereby if a router does not talk directly to the destination it checks with all of its direct peers to see if they do. As a result, data are duplicated as a side effect of access affording organic replication and scalable access. IPFS servers speak HTTP and therefore any data can be accessed in a browser or with a few lines of code from standard bioinformatics analysis tools (eg, cBio, Galaxy, and Jupyter).

### PatientExploreR-Cancer Gene Trust: Data Visualization

To facilitate interaction with CGT, we adapted a visualization application to browse, search, visualize, and download the clinical and genomic data shared on CGT. This application, called PatientExploreR-CGT, is adapted from our original PatientExploreR version [37]. PatientExploreR-CGT automatically pulls and maps all data from CGT into a user-friendly dashboard. This application is built in R (version 3.4.1) using the Shiny [38] (version 1.0.5) framework and directly interfaces with OMOP-formatted (version 5 or later) EHR data. In the front-end, the following Shiny-related packages are utilized: shinyWidgets [39], shinyjs [40], shinyalert [41], shinycssloaders [42], shinyBS [43], and shinythemes [44]. Visualizations were created using the plotly [45] and timevis [46] packages. In its backend, PatientExploreR-CGT makes use of ROMOP [47] to automatically extract and map pertinent concepts across all relevant tables (eg, person, observation, and condition occurrence). Data processing and manipulation were facilitated by data.table [48], DT [49], rjson [50], and dplyr [51]. This app can be freely accessed [52].

## Results

### Cancer Gene Trust Pilot Study

We provide the demographics of the pilot cohort in Table 2. In our cohort, the breakdown of primary cancer was as follows: seven with pancreatic adenocarcinoma, four with cholangiocarcinoma, and one each with anal squamous carcinoma, gastric cancer, colon cancer, gastrointestinal stromal tumor, cecal cancer, and metastatic cancer of unknown primary origin. An additional patient also had metastatic cancer of unknown primary origin but without EHR data. We provide a breakdown of all such data by patient and modality in Table 3.

**Table 2:** Cohort demographics and clinical information. Demographic breakdown of clinical pilot cohort.

Modality		Value
**Gender, n (%)**		
	Male	6 (33)
	Female	12 (67)
**Race, n (%)**		
	White	11 (61)
	Asian	5 (28)
	Unknown	2 (11)
**Ethnicity, n (%)**		
	Hispanic/Latino	2 (11)
	Not Hispanic/Latino	16 (89)
**Status, n (%)**		
	Alive	15 (83)
	Deceased	3 (17)
Age (years), mean (SD)		59.3 (13.3)

**Table 3:** Breakdown of Cancer Gene Trust data by patient and modality.

CGT^a^	Clinical		Genomics		Imaging		OMOP^b^ data breakdown		
CGT Public UUID^c^	Registry	OMOP	FMI^d^	UCSF^e^ 500	CT^f^	Pathology	Conditions	Procedures	Drugs
f9b6a782-bbf5-4be8-bf7e-d1a9586d9552	✓	✓	✓	N/A^g^	N/A	N/A	1597	1190	3661
c2e2e081-4c39-4201-8a27-7b469ed39490	✓	✓	✓	N/A	✓	✓	1350	969	2088
db2d85aa-4f94-4e77-8755-6b94a710c1aa	✓	✓	✓	N/A	✓	✓	2389	1394	3086
2fbc25da-3965-49c4-866f-72cf0abc2417	✓	✓	✓	N/A	N/A	N/A	930	654	1174
940171e7-d358-463a-8d9a-2b2fa90c2a84	✓	✓	✓	N/A	N/A	N/A	1179	624	1388
f0314175-2d19-4146-8754-fc5aed3ab420	✓	✓	✓	N/A	N/A	N/A	511	405	549
c7dbcfac-37ea-43f8-8899-1a9f2fb56341	✓	✓	✓	N/A	N/A	N/A	216	114	184
ef5c3164-6f45-4d3a-88f0-4509226c5571	✓	✓	N/A	✓	N/A	N/A	51	14	57
ec3d977b-c310-4df3-a444-f79bc3dd8b58	✓	✓	N/A	✓	N/A	N/A	811	505	776
131cf62d-ad78-49c1-a699-5bcc1004cd12	✓	✓	✓	N/A	N/A	N/A	155	42	110
cf11c31c-f4c3-48ba-9c46-66f406d0b7a1	✓	✓	✓	N/A	N/A	N/A	311	162	131
ccc2ba97-912f-4b62-b767-cca129ee6a56	✓	✓	N/A	✓	N/A	N/A	51	10	60
104ec531-5d95-41e2-ac72-f6cff2006b8e	✓	✓	✓	N/A	N/A	N/A	36	10	17
a5627ac3-450d-4036-ade8-99ae62a5c232	✓	✓	N/A	✓	N/A	N/A	857	439	805
5189efbe-3382-4353-ad2f-9afd0255c2c8	✓	✓	✓	N/A	N/A	N/A	875	276	674
253f0e2d-bebd-464b-81c5-8dd8385192b3	✓	✓	N/A	N/A	N/A	N/A	117	116	217
d199cfb0-91e8-471d-b1b3-53189cd64ee0	✓	✓	✓	N/A	✓	N/A	21	11	81
5d3205a3-28c4-45eb-bfd8-b32d67c3be0f	N/A	N/A	N/A	N/A	N/A	N/A	N/A	N/A	N/A

^a^CGT: Cancer Gene Trust.

^b^OMOP: Observational Medical Outcomes Partnership.

^c^UUID: universally unique identifier.

^d^FMI: Foundation Medicine.

^e^UCSF: University of California, San Francisco

^f^CT: computed tomography.

^g^N/A: not applicable.

### Breakdown of Available Data in Cancer Gene Trust by Patient

The CGT Public ID refers to the globally unique hexadecimal identifier per patient. ✓ indicates that data are available for that particular modality per patient. For the OMOP data, the numbers reflect how many data elements are available per modality.

### Genomic Breakdown of Cancer Gene Trust Cohort of Patients With Foundation One Reports

**Figure 2:**
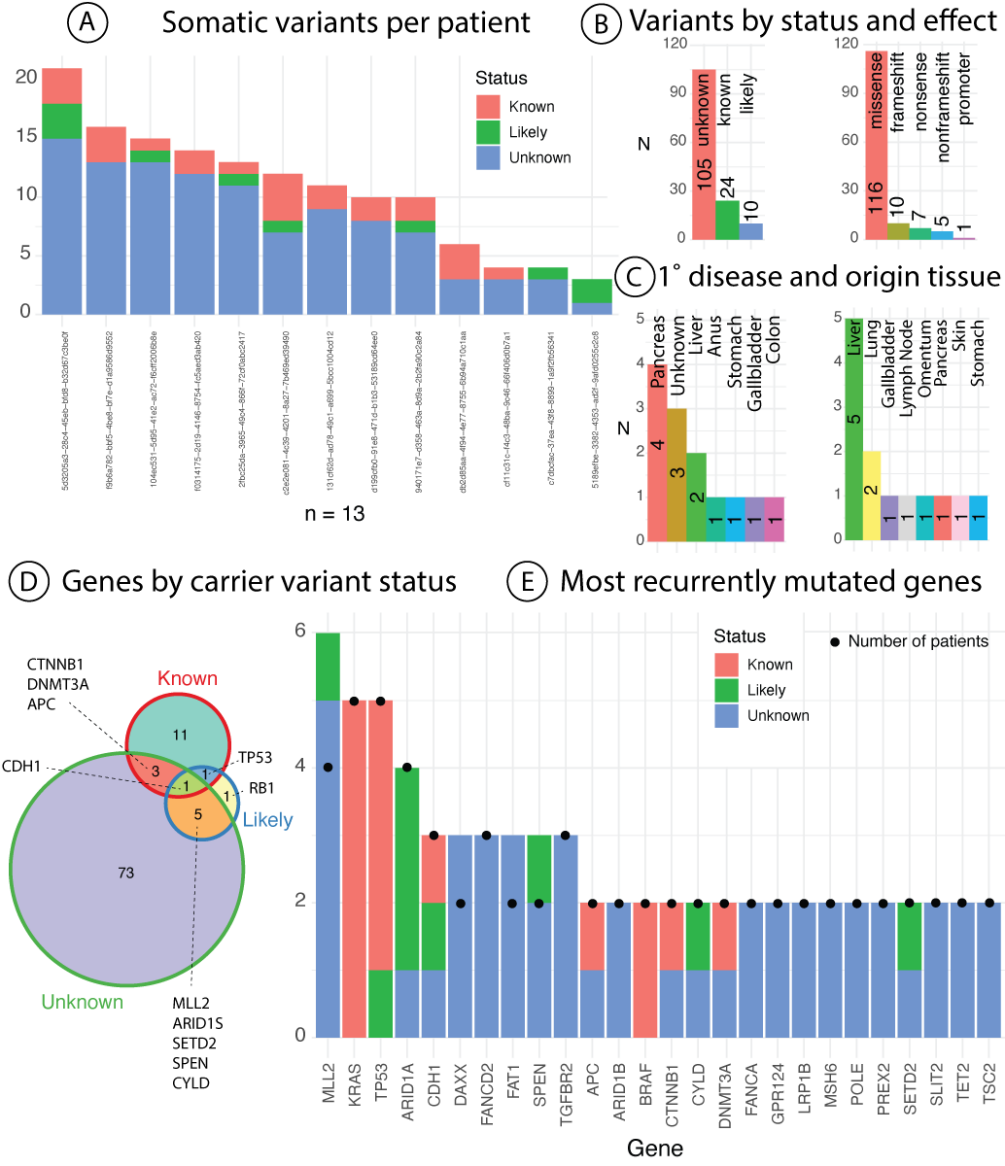
Breakdown of Foundation One genomics results for pilot cohort. (A) The breakdown of number of variants reported per patient stratified by their current knowledge status. (B) Breakdown of all variants for patients by effect (left) current knowledge status (right). (C) Distribution of cancer type per patient by primary disease (left) and tissue of origin (right). (D) Distribution of genes based on the current knowledge status of encompassed variants. (E) List of the most commonly recurrently mutated genes (N>1) by number of encompassed variants by status. Black dots represent number of unique patients with a variant in the gene.

Of patients with genomic data, the majority (n=13) had Foundation One sequencing performed and, as such, we focus on these data for a breakdown analysis (Figure 2). Across all patients, we identified 139 mutations in 95 genes (Multimedia Appendix 1). On average, patients had 10.69 (SD 5.34) somatic variants, with the most being 21 and the fewest being 3, across different current knowledge status (ie, known pathogenic, likely pathogenic, or of unknown consequence; panel A). On average, these somatic variants were primarily unknown (panel B left), with a mean of 8.07 (SD 4.57) per patient. Patients had an average of 2.18 (SD 0.98) of known and 1.43 (SD 0.79) likely variants. In terms of their functional effect, the majority of variants were missense (83.5% (116/139), panel B right). These patients had various primary diseases, the most prevalent being pancreatic (n=4, panel C left). For these patients, biopsies were taken from various tissues of origin, the most prevalent being liver (n=5, panel C right). Please refer to Multimedia Appendix 1 for a diagram illustrating connections between tissue of origin and primary disease for these patients. We further break down the functional effect and status of variants by tissue of origin and primary disease in Multimedia Appendix 1. These, of course, should be considered in context to the number of patients by tissue of origin and primary disease. With these considerations, we still found some interesting trends. For instance, lymph node tissue of origin (n=1 patient) had the fewest variants (n=3) with no known pathological variants, whereas omentum tissue of origin had the most for a single patient (n=21) with three known pathological variants. Of course, these trends could depend on patient-specific or severity variations, and will require more patient data.

Across all patients, the 95 genes contained variants with various levels of knowledge status, including overlapping domains if there were more than one variant identified per gene (panel D). Here, we also see that the majority of genes had variants of unknown significance (n=82). There were 10 genes that contained multiple variants of different knowledge classes. The most commonly mutated genes across individuals (Multimedia Appendix 1 and panel E, black dots) were KRAS and TP53 (n=5) followed by ARID1A and MLL2 (n=4). In a similar vein, MLL2 has the most unique variants identified (n=6 variants across 4 patients), followed by KRAS and TP53 (n=5 variants across 5 patients). The majority of genes with more than one carrier contained variants of unknown significance only (54% (14/26)), further exemplifying the need for combining real-world EHR with such genomic data. We further visualize the landscape of variants of unknown significance by effect overall and on a per-patient level in Multimedia Appendix 1. We notice that for 1 patient with pancreatic cancer and tissue biopsy, for instance, there is one nonsense mutation in TGFBR2 with a current unknown knowledge status. According to TCGA GDC data portal, there are only 15 cases of primary site pancreatic cancer (TCGA-PAAD) with variants in this gene, and only two are stop-gain. Sharing data such as these with other researchers could quickly expand current knowledge status of variants and their association with disease.

### Comparing Robustness of Clinical Data Procedures

To identify the most robust format of clinical data to share on CGT, we assessed whether there was a significant difference in scoring quality between two disparate data formats, specifically the prospectively collected registry and retrospectively gathered OMOP. We hypothesized that there would be no overall difference in scoring quality because both methodologies in theory should capture the main core competencies of interest.

Although we found that total score across all patients and data elements were higher for registry compared with OMOP (Table 4; 642 vs 560), this difference was not statistically significant (*P*=.13, V=44).We further analyzed any significant discrepancies by core competency data element (Multimedia Appendix 1; Table 1 for element descriptions and source).We found no significant difference for Gender (*P*=.35, V=3), Ethnicity (*P*=.17, V=6), Race (*P*=.17, V=13), Year of Birth (*P*=.35, V=3), Basis of Diagnosis (*P*=.66, V=45), Cancer Site (*P*=.09, V=0), Therapeutic Agent/Modality (*P*=.17, V=21), and Beginning and End Dates of Treatment (*P*=.47, V=20). We did find, however, that there was a significant difference between OMOP and registry scoring for Date of Diagnosis (*P*=.004, V=0), with registry having higher scores (*P*=.002, V=0), and Cancer Histology (*P*=.0004, V=0), with registry having higher scores (*P*<.001, V=0). See Multimedia Appendix 1 for per patient, per element scores for registry and OMOP, respectively.

Break down of *gold standard* elements and their respective fields in registry and OMOP is given in Table 4.

**Table 4:** Overall patient scores for registry vs Observational Medical Outcomes Partnership formats.

Patient	Registry	OMOP^a^
f9b6a782-bbf5-4be8-bf7e-d1a9586d9552	39	28
c2e2e081-4c39-4201-8a27-7b469ed39490	41	34
db2d85aa-4f94-4e77-8755-6b94a710c1aa	42	32
2fbc25da-3965-49c4-866f-72cf0abc2417	48	30
940171e7-d358-463a-8d9a-2b2fa90c2a84	31	41
f0314175-2d19-4146-8754-fc5aed3ab420	29	39
c7dbcfac-37ea-43f8-8899-1a9f2fb56341	15	33
ef5c3164-6f45-4d3a-88f0-4509226c5571	50	29
ec3d977b-c310-4df3-a444-f79bc3dd8b58	35	33
131cf62d-ad78-49c1-a699-5bcc1004cd12	35	33
cf11c31c-f4c3-48ba-9c46-66f406d0b7a1	47	29
ccc2ba97-912f-4b62-b767-cca129ee6a56	13	33
104ec531-5d95-41e2-ac72-f6cff2006b8e	35	24
a5627ac3-450d-4036-ade8-99ae62a5c232	45	34
5189efbe-3382-4353-ad2f-9afd0255c2c8	47	38
253f0e2d-bebd-464b-81c5-8dd8385192b3	46	37
d199cfb0-91e8-471d-b1b3-53189cd64ee0	44	33
Total	642	560

^a^OMOP: Observational Medical Outcomes Partnership.

Total score per patient per data modality, specifically registry vs OMOP, compared with *gold standard* raw EHR data. Each score is the sum of all elements analyzed. Patient scores broken down by element can be found in Multimedia Appendix 1.

### Developing a Clinical Narrative From Cancer Gene Trust Data

Although safely, securely, and robustly sharing clinically related patient data is an important procedure in and of itself, we want to demonstrate the power of this framework by compiling a clinical narrative solely from data shared on CGT. We elected to use patient c2e2e081-4c39-4201-8a27-7b469ed39490 as a highlighted example (see Multimedia Appendix 1 for all relevant CGT hash information for this patient). We further show how to identify these data points using PatientExploreR-CGT in the following section.

On Day 1 (26,346 days from birth), patient underwent laparoscopic cholecystectomy (at a prior institution) which confirmed moderately differentiated adenocarcinoma with mucinous features. On day 42 (26,387), pathology was reviewed at UCSF which confirmed stage at pT2Nx. On Day 75 (26,420), patient underwent open partial hepatectomy, portal lymphadenectomy, and appendectomy. An FNA of RUQ skin nodule at prior trochar site on Day 195 (26,540) identified adenocarcinoma consistent with recurrence/metastasis from primary gallbladder site. CT C/A/P on Day 196 (26,540) showed multiple new peritoneal and ventral abdominal wall soft tissue nodules suspicious for metastases.

**Figure 3:**
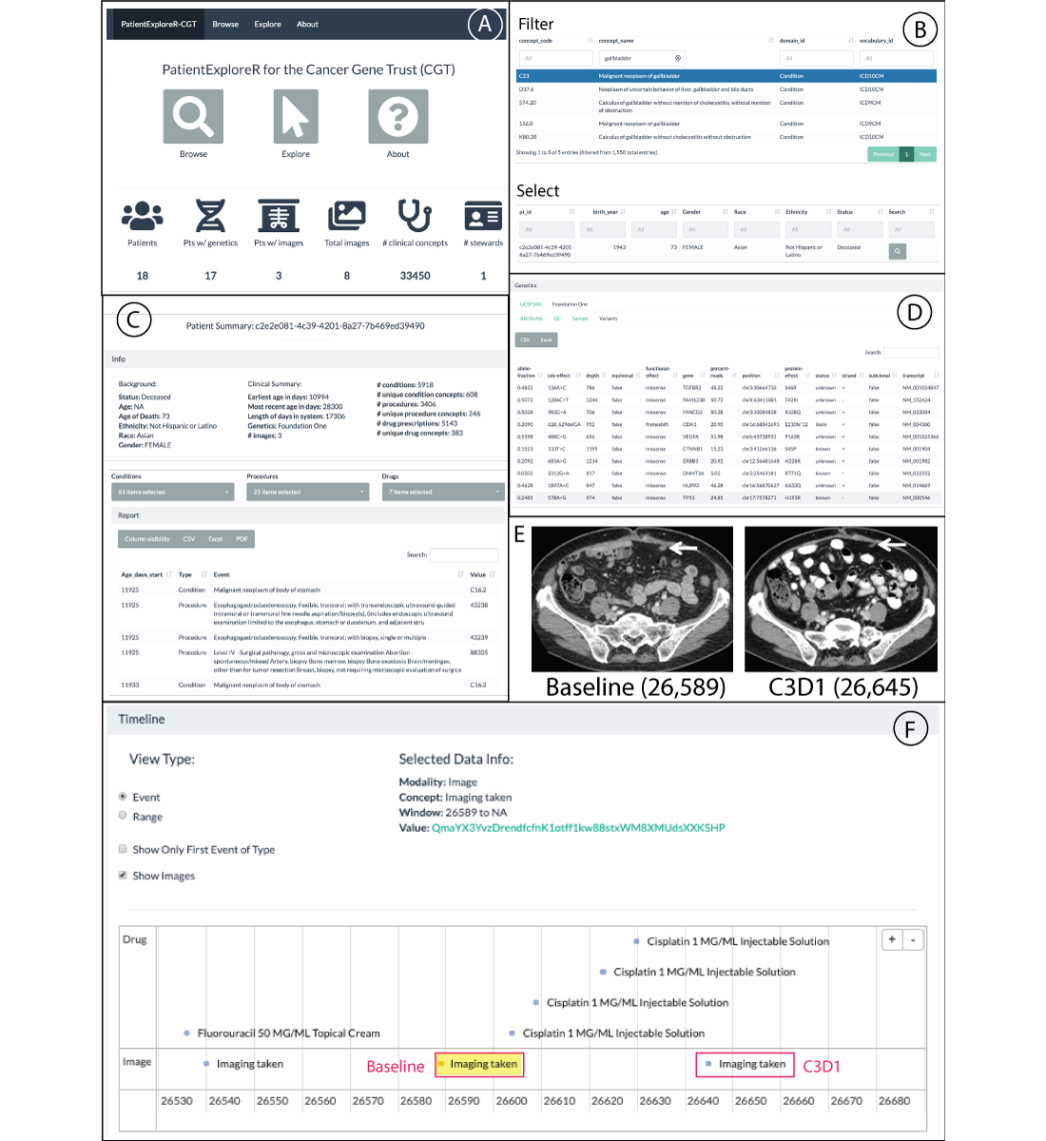
PatientExploreR visualization app for Cancer Gene Trust (CGT). Breakdown of features available for the public PatientExploreR visualization application for CGT. (A) Landing page which has all options for browsing patients, exploring patient data, and information about CGT. This page also displays the amount of data currently compiled on CGT. (B) Browse tab for filtering for patient based on clinical concepts and/or demographics. This list is filtered by Gallbladder-related disease and patient c2e2e081-4c39-4201-8a27-7b469ed39490 was selected. (C) Explore tab that details patient’s background and clinical summary. The user can interact with clinical, genomic, and imaging data for selected patient. These data can be filtered and exported and control what is shown visually in the timeline view below. (D) Genomics data extracted and displayed from either the Foundation One or UCSF 500 report. (E) Two sample image scans that can be found via the imaging submenu or from clicking within the timeline view below. These CT scans show baseline and C3D1, 26,589 and 26,645 age in days, respectively. Baseline contrast enhanced CT of the abdomen shows a peritoneal metastasis (arrow) measuring 12×8 mm. Posttreatment contrast enhanced CT of the abdomen shows decreased size of the peritoneal metastasis (arrow) measuring 10×6 mm. (F) Timeline view of selected clinical and image data. This timeline view was produced by selecting the associated relevant medications in the Drug pull-down menu, specifically: capecitabine, cisplatin, fluorouracil, and trastuzumab. With Show Images selected, we can see what relevant medications the patient was prescribed when the images were taken. Pressing the link next to Value above, the user will be directed to all images taken during that visit, which can be viewed on an appropriate (eg, DICOM viewer) browser.

Patient signed informed consent for CC#16457 clinical trial on Day 238 (26,583) andcompleted baselinescans on Day 244 (26,589; Figure 3E [left]; hash ids: QmaYX3YvzDrendfcfnK1otff1kw88stxWM8 XMUdsXXKSHP [parent], Qmd7V8hS2mCtup RLYk6Qm2AMHyk6X7Y4QPTDqZe7UCUnUT [image]) which showed unchanged disease from Day 196. Patient randomized to Arm B: merestinib/placebo + cisplatin + gemcitabine (not available in OMOP data) on Day 257 (26,602) and completed Cycle 1, Day 1 cisplatin + gemcitabine on Day 260 (26,605). On Day 286 (26,631), Cycle 2, Day 8 cisplatin + gemcitabine was completed.

On Day 300, a CT C/A/P was performed (26,645; Figure 3E [right]; hash ids: QmQ6PtwhTM qw9b3SFsa1qfW79kGK7tPrhrUHpKVLtxmj1i [parent], QmZmVEsqNeCDuzUDDvLWYUdbxQ2QZ ehDhkdzyCNvX8gFJF [image]) and showed stable scattered abdominal wall, peritoneal and retroperitoneal implants. Interval progression of mild intrahepatic biliary dilatation, possibly due to new soft tissue prominence at the porta hepatis, concerning for recurrence. However, unchanged small upper lobe pulmonary nodules were noted and stable disease was concluded per RECIST, with 18.18% decrease in sum of target lesion diameters.

### Exploring Cancer Gene Trust Data on PatientExploreR-Cancer Gene Trust

To further operationalize the CGT framework, we adapted an application called PatientExploreR to seamlessly interface with CGT to effectively explore, visualize, and download the data. We envision this application to be particularly useful for individuals without much data extraction and manipulation experience. This application requires no registration and is publicly available [52]. PatientExploreR-CGT pulls all OMOP data from CGT, maps all clinical concepts according to the CDM, and provides convenient links to genomic data as well as image data in the context of their clinical history. In Figure 3, we demonstrate the power of the visualization by showing a detailed timeline of the above patient’s treatment timeline around the time of the available CT scans.

## Discussion

### Overview

In this study, we have consented patients in an IRB-approved process to share deidentified EHRs, genomic, and imaging data using a blockchain-authenticated framework called CGT. Our goal of this pilot study was to demonstrate the process of patient consent to data sharing within a large public health institution as well as to create a framework that can facilitate other institutions, physicians, and patients to add their own data. The benefit of a block-chain authenticated system was more geared to decentralized access (authorization) rather than privacy or security (authentication) as all submissions are public by design. As we hypothesized, all 18 patients in the pilot study did not have reservations about sharing their data, which has been similarly demonstrated [9], and we believe patients from other institutions have similar beliefs. Patient privacy was a top priority for this project and we actively coordinated the highest-standards for deidentification processing of all data shared (see Multimedia Appendix 1 for deidentification process).

In designing the CGT, we had to overcome the existing challenges in this space, namely that this framework should be secure, efficient, and scalable while being cost-efficient, open to the public, and not owned by a single institution. We also had to determine not only which data should be shared but also the appropriate format of such data that would balance interoperability with speed of sharing. Our pilot also addresses cultural and institutional challenges, both perceived and real, including the IRB, patient consent and education, and other elements.

CGT is designed as an alternate approach to centralized data repository platforms such as Medical Information Mart for Intensive Care [53] which have enabled a slew of powerful research. Unlike these primarily static databases, CGT can facilitate rapid and continual data being shared from the clinical care system as close to the time of generation and extraction as possible. Both systems have their merits and hopefully they will be complementary in providing access to deidentified EHR data to enable personalized medicine. Furthermore, CGT enables researchers to use and interpret medical data instead of resolving disparate access methods from multiple sources or failing entirely because data are simply not available in any format. Indeed, it is our hope that CGT can facilitate research studies and enhance clinical care on a timescale not previously possible, while allowing data holders to maintain the privacy and security of individual data sources and the nonpublic subset of the data [36]. At the same time, this entire process will respect individual patient consents and cultural data sharing preferences and expectations. CGT enables aggregation of data from all consenting patients. CGT might bolster cancer research and help physicians, patients, payers, and other stakeholders make more informed decisions about the increasingly complex diagnosis and treatment of cancer as well as its reimbursement. CGT functions as a bridge between the highly regulated HIPAA environment (Figure 1) and the open World Wide Web internet environment. To alleviate concerns about data ownership, CGT is built on a decentralized, democratized blockchain format and will remain free and open.

### Principal Findings

Compared with a list of *gold standard* data elements [11] that should be shared in such a project, we found that there was no significant difference in completeness between a prospectively collected registry and a retrospective (OMOP) procedure for clinical data. Certain data elements, however, were more robustly recorded in the registry format, specifically Date of Diagnosis and Cancer Histology. For analyses that aim to further personalized medicine, such pieces of information might be critical, and we hope the findings from this study can help improve the continually adapting OMOP model to better encode such information. These lapses could also be due to institution-specific extract, transform, load (ETL) procedures.

Each strategy has its respective benefits and weaknesses. Because registry data are manually coded, specific key pieces of information can be easily highlighted and identified. Furthermore, for registry data to be submitted to SEER, all pieces of information must be detailed, but this process is manual and time consuming, and often results in different stages of aggregation per patient. As such, we found higher levels of variance in registry records compared with those in OMOP (mean 37.77, SD 10.87 vs mean 32.94, SD 4.26), which could reflect delays in manual data aggregation (ie, *suspense* states) or quality. It was clear though that more patients had more complete information from registry data than OMOP, with 5 patients having more than 90% completeness cores (ie, >45 total score) in registry vs 0 in OMOP. However, by relying on the open source OMOP standard, instead of registry or a proprietary EHR structure, the barrier for distributing and sharing data is drastically lowered through reducing ETL transformation, which also lowers cost through leveraging the conversion processes already occurring in many hospital systems. Researchers recently demonstrated the power of OMOP for facilitating phenotype transfer across sites [54], which aligns well with the goal of CGT. The additional costs of time are the clinical and regulatory tasks involved in consenting patients and obtaining, anonymizing, and uploading data. This process accounts for the majority of cost which will further decrease in high volume.

### Limitations

There are many limitations of this study that need to be addressed. Both the registry and OMOP EHR extract did not contain all valuable and relevant core data elements. Therefore, the comparison of data robustness cannot be extended to all *gold-standard* elements that ideally should be shared in such a project. As OMOP is from retrospective extraction process, there is no immediate way to automatically identify primary cancer and therapeutic efficacy, although we hope this can be mediated by subsequent incorporation of deidentified notes or new schema adaptations or developments. Similar to any noncurated database, data quality for both registry and OMOP is limited by those who entered it and could be affected by infrastructural biases of individuals and EHR systems [55]. In addition, the current framework is steward based, which means that there needs to be a single individual or team representative to submit data per institution. Similar to any cross-institution data link of deidentified data, there is no procedure in place to be able to map the same patient across stewards as there exists within the registry system. Although we tried to create a rule-based scoring system that is as unbiased as possible involving 2 separate reviewers, the manual scoring of data elements did contain levels of subjectivity and potential ambiguity, which is fully detailed in the Multimedia Appendix 1.

There are also risks of reidentification associated with data sharing, even beyond accidental leakage. Even for incomplete, fully deidentified data, for instance, a recent study was able to use generative copula-based method to accurately reidentify 99.98% of American individuals based on only 15 demographic attributes [56]. Of course, many of these variables used in this paper are not available in this dataset, but it is important to note as other models might be developed in the future those could be applied to the data shared. Overall, these risks need to be weighed against the stagnation associated with keeping these valuable data siloed. Not sharing all details pertaining to treatment efficacy and adverse drug effects are not in the best interests of general public and overall scientific and medical community. Despite these limitations, open scientific data sharing has been an enormous boon in many fields and we believe that CGT presents a proof of concept that useful medical data can be openly shared. We further demonstrated the feasibility and utility of this process in a pilot study and provide fully detailed steps for other institutions to consent and add their patients’ data. The ultimate success of this platform will be determined by the flow of patient data and how it can be used to facilitate discoveries and help personalize treatment.

### Conclusions

Each cancer case is unique and requires as much data as possible to inform ideal treatment decisions. The more data that exist and are released can help clinicians identify ideal personalized treatment for their patients. We found the OMOP CDM is a scalable format for dissemination, although it can be improved by better information in key data element fields such as cancer histology as compared with a prospectively collected registry format. The OHDSI Oncology Working Group [12] is currently developing an extension to OMOP to support observational cancer research that better captures and records elements we found available in the registry format but not in the current OMOP implementation. We believe such an effort is invaluable to reconcile these differences and should be integrated into the future version of CGT. Put together, we hope that the CGT framework, pilot study, and interactive visualization application furthers the ideals of the cancer Moonshot project, unleashing data trapped in silos to further cancer research and reveal patterns that can help further personalize treatment.

## Appendix

Multimedia Appendix 1 Supplementary materials, including supplementary methods, figures, and tables.

## Abbreviations

CDM: common data model
CGT: Cancer Gene Trust
CT: computed tomography
EDW: Enterprise Data Warehouse
EHR: electronic health record
ETL: extract, transform, load
HIPAA: Health Insurance Portability and Accountability Act
IPFS: InterPlanetary File System
IRB: institutional review board
MRN: medical record number
OHDSI: Observational Health Data Sciences and Informatics
OMOP: Observational Medical Outcomes Partnership
PHI: protected health information
SEER: Surveillance, Epidemiology, and End Results
UCSF: University of California, San Francisco
UUID: universally unique identifier
