# Supplementary material for: Blockchain-Authenticated Sharing of Genomic and Clinical Outcomes Data of Patients With Cancer: A Prospective Cohort Study
Source: J Med Internet Res. 2020 Mar 20;22(3):e16810. doi: 10.2196/16810 (PMC7125440; doi:10.2196/16810)
Supplement: Multimedia Appendix 1 [file jmir_v22i3e16810_app1.docx]

**SUPPLEMENTARY MATERIALS**

**Blockchain-Authenticated Sharing of Genomic and Clinical Outcomes Data of Patients With Cancer: a Prospective Cohort Study**

Benjamin S. Glicksberg^1,2,3+^, Shohei Burns^4+^, Rob Currie^5^, Ann Griffin^6^, Zhen Wang^7^, David Haussler^5,8^, Theodore Goldstein^1,5^, Eric A. Collisson^4*^

^+^Authors contributed equally

^*^Correspondence: Eric A Collisson, MD; collissonlab@gmail.com; (415) 353-9888

**Affiliations**

1. Bakar Computational Health Sciences Institute, University of California, San Francisco, San Francisco, US.
2. Hasso Plattner Institute for Digital Health at Mount Sinai, Icahn School of Medicine at Mount Sinai, New York, US.
3. Department of Genetics and Genomic Sciences, Icahn School of Medicine at Mount Sinai, New York, US.
4. Department of Medicine, Division of Hematology and Oncology, University of California, San Francisco, San Francisco, US.
5. UC Santa Cruz Genomics Institute, University of California, Santa Cruz, Santa Cruz, US.
6. Helen Diller Family Comprehensive Cancer Center, University of California, San Francisco, San Francisco, US.
7. Department of Radiology and Biomedical Imaging, University of California, San Francisco, San Francisco, US.
8. Howard Hughes Medical Institute, Santa Cruz, US.

**Supplementary Methods**

***De-identification Process***

The definition of what constitutes identified vs. de-identified data is sharply debated. A conservative view is that identified data results from any combination of facts that could allow a determined researcher (such as a marketing organization) to re-identify an individual. For example, it may be possible to use a combination of prescriptions[1] to re-identify an individual by projecting a list of prescriptions through a pharmaceutical company marketing database through a data fusion of two databases and then to use this information to target marketing materials.  A more feasible safe harbor approach, which we selected, balances the benefits of data sharing against privacy and takes the view that only the HIPAA identifiers [2]are PHI and must be expunged from public, de-identified data sharing.

The complete IRB document can be found on the main web resource (<https://www.cancergenetrust.org/docs/cgt-ucsf-protocol.pdf>).

*Patient Consent*

Patient was fully consented by both the treating provider/Principal Investigator (PI; Eric Collisson) and the clinical research coordinator (CRC; Shohei Burns) present. The patient was given a copy of the consent form and reviewed every paragraph of the consent form with the PI and the CRC. The CRC and treating investigator specifically discussed the specific implications of this study, including the collection, de-identification, and sharing of medical data (including clinical, radiographic, and genetic data). The risks of data-sharing, including a potential breach of privacy, were also discussed at length and the patient thoroughly reviewed the Appendix which listed data that would and would not be shared as part of this study. The Patient Consent document can be found at <https://www.cancergenetrust.org/docs/cgt-ucsf-consent.pdf>.

*Images*

After consenting the patient, the CRC requested individual imaging data from UCSF’s PAX medical imaging system. These de-identified scans conformed to Supplement 142: Clinical Trial De-identification Basic Profile which removed any identifying PHI from the images as well as any accompanying metadata. These de-identified DICOM format files were downloaded onto a secure hard drive, checked manually for any remaining PHI, and forwarded electronically for submission to the CGT. Patient specific pathology slides were requested from the UCSF pathology department after patient consent was obtained. An Aperio scanner was used to physically scan and digitize the relevant pathology slides for each consented patient. No protected health information was included on the scanned slides including any accompanying metadata or burned-in information on the digitized files themselves. These scanned de-identified images were forwarded electronically for submission to the CGT.

*Genomics*

UCSF500

After consenting the patient, the CRC requested the UCSF500 VCF file with all lines except for those marked ‘SOMATIC’ removed and then forwarded this de-identified file to steward for submission to the CGT.

Foundation Medicine

After consenting the patient, the CRC requested from Foundation Medicine the machine readable XML file for the patients test (sample can be found here: <https://github.com/cancergenetrust/submit/tree/master/public/samples>). The CRC then dragged this file onto a client side signal page web application (https://cancergenetrust.github.io/submit/). This application read in the XML file, removed all sections except for the somatic variant data (rr:ResultsReport->rr:ResultsPayload->variant-report) as well as any patient identifiers (_attributes->specimen and attributes->test-request). The remaining data after transformation were exported into a json file that the CRC forwarded to be submitted to the CGT. The original XML files were only processed on the CRC’s computer in their browser therefore none of the data before de-identification was ever sent over the internet.

*Clinical Electronic Health Record (EHR) data*

Registry

After consenting a patient, the CRC requested from the registry representative at UCSF (Ann Griffin) an export of the patients’ registry extract from the CNEXT database in Excel format (sample can be found here: <https://github.com/cancergenetrust/submit/tree/master/public/samples>). The CRC then dragged this file onto a client side signal page web application (<https://cancergenetrust.github.io/submit/>) that performed the following transformations using a transformation table (<https://github.com/cancergenetrust/submit/blob/master/public/clinicalFilter.tsv>):

- Removed all fields except those covered in the consent (Fields with an entry under the CGT column)
- Transformed any of the remaining fields if indicated (see Transform column). Specifically, all dates were transformed to durations as days from first contact.

The remaining data after transformation were exported into a json file that the CRC saved to their disk (seer.json) and forwarded to the steward to be submitted to the CGT. The original excel file was only processed on the CRC’s computer in their browser therefore none of the data before de-identification was ever sent over the internet.

OMOP

The Enterprise Data Warehouse (EDW) Academic Research Systems at UCSF (<https://it.ucsf.edu/about/teams/academic-research-systems>) at UCSF is a data warehouse that is responsible for many EHR-related tasks, including extraction and de-identification. We contracted EDW to perform an extraction and de-identification of OMOP data for patients in our cohort.

EDW correspondence timeline:

9/19/18: Ticket opened requesting extraction for identified and de-identified OMOP data: RITM0155713 (PI: Goldstein). Identified data was requested so we could verify the scope and types of data available pertaining to our variables of interest.

- In accordance with IRB, we initially requested data from the following OMOP tables:
  - person
  - death
  - visit_occurrence
  - condition_occurrence
  - procedure_occurrence
  - drug_exposure
  - observation
- It was difficult to ascertain which specific items in these tables reflected cancer-related information, but the scope of the IRB allowed for information pertaining to other health data

9/20/18: Statement of Work provided by EDW for review

9/25/18: Call with EDW to discuss nuances of tasks

- We uploaded a list of relevant columns for which we request data.
- EDW to review and indicate PHI-related fields

9/26/18: EDW provides initial review for relevant column data

9/28/18: Discussion to refine requested columns for OMOP data

10/1/18: Confirmation on both ends pertaining to acceptable columns for extraction and marking which columns with PHI will have to be scrubbed.

10/5/18: Confirmed with EDW that we will not be sharing identified data; only de-identified data outside of UCSF as reflected in our consent and IRB

- *Identified* data delivered to team for initial review

10/12/18: We confirmed that the data is suitable for our needs. EDW team begins working on de-identification task.

10/18/18: EDW reaches out to indicate that data have been de-identified. They are *undergoing a process for data peer review* before releasing to us.

- Some confusion pertaining to format of de-identified data release. Originally, EDW returned data with completely synthetic patient IDs. Unfortunately, because of this, we were not able to match the patients’ OMOP data to their CGT IDs and other de-identified data (i.e., genomic reports)

10/19/18: As a solution, we provided a crosswalk file that maps patient identifier to CGT ID. EDW to return de-identified data with CGT ID instead of synthetic ID.

10/22/18: EDW delivers de-identified OMOP data in CGT ID format

10/31/18: EDW and CGT team confirm that data is sufficient and in correct format. Ticket closed.

*Final internal review:*

04/11/19: Manually checked all fields and elected to remove “sig” field from drug_exposure table. Converted OMOP to json for submission.

**Final breakdown of OMOP data release:**

*Note: some columns have been modified for de-identification purposes and are not in original OMOP schema format*

person:

- year_of_birth
- ethnicity_concept_id
- gender_concept_id
- race_concept_id

death:

- death_in_days

drug_exposure:

- days_supply
- dose_uinit_source_value
- drug_concept_id
- drug_end_in_days
- drug_start_in_days
- drug_type_concept_id
- quantity
- refills

condition_occurrence:

- condition_concept_id
- condition_start_in_days
- condition_type_concept_id

procedure_occurrence:

- procedure_concept_id
- procedure_days
- procedure_type_concept_id

***Manual Scoring Rubric of Clinical Data Elements***

In order to determine the quality, accuracy, and completeness of the data elements in each data source, we compiled a list of key features [3] to compare against the raw EHR data from the patients’ charts. As this process can be subjective, we developed a rigorous process to remove as much bias as possible. All primary investigators agreed on the following rubric before any scoring took place. Two separate investigators then reviewed and scored each data element. A third investigator then additionally reviewed these results to confirm the findings and reconcile and discrepancies.

We designed a scoring system that ranged from 0-5 for each relevant clinical concept for OMOP and registry as compared to the raw EHR data. To further limit bias in terms of subjectivity in this procedure, we developed and used the following rubric as closely as possible:

5 points = 81-100% match to gold-standard. Awarded for perfect or near perfect match to gold-standard for single element. For two-date element fields, awarded for match to both dates. Examples for this scoring category: for Race, ‘Chinese’ and ‘Asian’ would both be coded as 5 since Race grouping will collapse to Asian. Non-Spanish was considered equivalent to Non-Hispanic/Latino.

4 points = 61-80% match to gold-standard. General concept match but not necessarily specific. An example of this scoring category: for Primary Site, ‘pancreas’ vs. ‘pancreas head’.

3 points = 41-60% match to gold-standard. Awarded for two-date field where one date is correct but the other is missing or incorrect. Another example for this scoring category would be if two therapeutic agents were provided out of the complete three drug regimen found in the raw EHR.

2 points =21-40% match to gold-standard. This scoring category was used in cases of incorrect or missing data for the majority of relevant data elements, such as when one therapeutic agent was given out of a complete three drug regiment from the raw EHR.

1 point = 1-20% match to gold-standard. Awarded when data for element is entered but are incorrect or are too general. An example for this scoring category would be for Race, where ‘Other’ or ‘Unknown’ are given but the “correct” answer is ‘White’.

0 points = Awarded for elements containing no data

**Supplementary Figures**

Supplementary Figure 1: Patient tissue of origin to primary disease relationship

Sannkey plot detailing relationship of tissue of origin to primary disease for each patient. The thinnest line (e.g., between Gallbladder) represents a single patient.

Supplementary Figure 2: Functional effect and status by tissue of origin

Breakdown of functional effect (A) and knowledge status (B) by tissue of origin. The number of patients for each tissue of origin is inidcated at the bottom of the plot.

Supplementary Figure 3: Functional effect and status by primary disease

Breakdown of functional effect (A) and knowledge status (B) by primary disease. The number of patients for each tissue of origin is inidcated at the bottom of the plot.

Supplementary Figure 4: Further breakdown of variants of unknown significance

Further visualization of all variants of unknown significance (n=105) by functional effect (A) and per patient (B).

**Supplementary Tables**

Supplementary Table 1: Foundation One breakdown of variants by patient

functional-effect gene status cgt_id

missense KRAS known db2d85aa-4f94-4e77-8755-6b94a710c1aa

missense CHEK1 known db2d85aa-4f94-4e77-8755-6b94a710c1aa

missense PREX2 unknown db2d85aa-4f94-4e77-8755-6b94a710c1aa

missense EP300 known db2d85aa-4f94-4e77-8755-6b94a710c1aa

missense DICER1 unknown db2d85aa-4f94-4e77-8755-6b94a710c1aa

missense CREBBP unknown db2d85aa-4f94-4e77-8755-6b94a710c1aa

missense PDCD1LG2 unknown 5189efbe-3382-4353-ad2f-9afd0255c2c8

frameshift ARID1A likely 5189efbe-3382-4353-ad2f-9afd0255c2c8

nonsense SPEN likely 5189efbe-3382-4353-ad2f-9afd0255c2c8

missense STK11 known 5d3205a3-28c4-45eb-bfd8-b32d67c3be0f

missense MLL2 unknown 5d3205a3-28c4-45eb-bfd8-b32d67c3be0f

missense ERBB2 unknown 5d3205a3-28c4-45eb-bfd8-b32d67c3be0f

missense KRAS known 5d3205a3-28c4-45eb-bfd8-b32d67c3be0f

missense TP53 known 5d3205a3-28c4-45eb-bfd8-b32d67c3be0f

missense APC unknown 5d3205a3-28c4-45eb-bfd8-b32d67c3be0f

nonframeshift FBXW7 unknown 5d3205a3-28c4-45eb-bfd8-b32d67c3be0f

missense KEAP1 unknown 5d3205a3-28c4-45eb-bfd8-b32d67c3be0f

frameshift ARID1A likely 5d3205a3-28c4-45eb-bfd8-b32d67c3be0f

frameshift SETD2 likely 5d3205a3-28c4-45eb-bfd8-b32d67c3be0f

missense FAT1 unknown 5d3205a3-28c4-45eb-bfd8-b32d67c3be0f

missense TGFBR2 unknown 5d3205a3-28c4-45eb-bfd8-b32d67c3be0f

missense CHEK2 unknown 5d3205a3-28c4-45eb-bfd8-b32d67c3be0f

nonsense RB1 likely 5d3205a3-28c4-45eb-bfd8-b32d67c3be0f

missense CD79B unknown 5d3205a3-28c4-45eb-bfd8-b32d67c3be0f

missense PRDM1 unknown 5d3205a3-28c4-45eb-bfd8-b32d67c3be0f

missense CDH1 unknown 5d3205a3-28c4-45eb-bfd8-b32d67c3be0f

missense HNF1A unknown 5d3205a3-28c4-45eb-bfd8-b32d67c3be0f

missense EMSY unknown 5d3205a3-28c4-45eb-bfd8-b32d67c3be0f

missense SLIT2 unknown 5d3205a3-28c4-45eb-bfd8-b32d67c3be0f

missense RANBP2 unknown 5d3205a3-28c4-45eb-bfd8-b32d67c3be0f

missense TGFBR2 unknown c2e2e081-4c39-4201-8a27-7b469ed39490

missense FAM123B unknown c2e2e081-4c39-4201-8a27-7b469ed39490

missense FANCD2 unknown c2e2e081-4c39-4201-8a27-7b469ed39490

frameshift CDH1 likely c2e2e081-4c39-4201-8a27-7b469ed39490

missense VEGFA unknown c2e2e081-4c39-4201-8a27-7b469ed39490

missense CTNNB1 known c2e2e081-4c39-4201-8a27-7b469ed39490

missense ERBB3 unknown c2e2e081-4c39-4201-8a27-7b469ed39490

missense DNMT3A known c2e2e081-4c39-4201-8a27-7b469ed39490

missense NUP93 unknown c2e2e081-4c39-4201-8a27-7b469ed39490

missense TP53 known c2e2e081-4c39-4201-8a27-7b469ed39490

nonframeshift PIK3CA known c2e2e081-4c39-4201-8a27-7b469ed39490

missense DAXX unknown c2e2e081-4c39-4201-8a27-7b469ed39490

missense MAGI2 unknown f9b6a782-bbf5-4be8-bf7e-d1a9586d9552

missense MLL2 unknown f9b6a782-bbf5-4be8-bf7e-d1a9586d9552

missense LRP1B unknown f9b6a782-bbf5-4be8-bf7e-d1a9586d9552

missense CTNNB1 unknown f9b6a782-bbf5-4be8-bf7e-d1a9586d9552

missense PTCH1 known f9b6a782-bbf5-4be8-bf7e-d1a9586d9552

missense SNCAIP unknown f9b6a782-bbf5-4be8-bf7e-d1a9586d9552

nonsense APC known f9b6a782-bbf5-4be8-bf7e-d1a9586d9552

missense CTNNA1 unknown f9b6a782-bbf5-4be8-bf7e-d1a9586d9552

missense KEL unknown f9b6a782-bbf5-4be8-bf7e-d1a9586d9552

missense GPR124 unknown f9b6a782-bbf5-4be8-bf7e-d1a9586d9552

missense MLL2 unknown f9b6a782-bbf5-4be8-bf7e-d1a9586d9552

missense ARID1A unknown f9b6a782-bbf5-4be8-bf7e-d1a9586d9552

missense CDK4 unknown f9b6a782-bbf5-4be8-bf7e-d1a9586d9552

missense BRCA2 unknown f9b6a782-bbf5-4be8-bf7e-d1a9586d9552

missense KRAS known f9b6a782-bbf5-4be8-bf7e-d1a9586d9552

missense FANCD2 unknown f9b6a782-bbf5-4be8-bf7e-d1a9586d9552

missense ALK unknown 2fbc25da-3965-49c4-866f-72cf0abc2417

missense FANCA unknown 2fbc25da-3965-49c4-866f-72cf0abc2417

missense CSF1R unknown 2fbc25da-3965-49c4-866f-72cf0abc2417

frameshift ARID1A likely 2fbc25da-3965-49c4-866f-72cf0abc2417

missense FAT1 unknown 2fbc25da-3965-49c4-866f-72cf0abc2417

missense ATM unknown 2fbc25da-3965-49c4-866f-72cf0abc2417

missense FAT1 unknown 2fbc25da-3965-49c4-866f-72cf0abc2417

frameshift ARID1B unknown 2fbc25da-3965-49c4-866f-72cf0abc2417

missense KIT unknown 2fbc25da-3965-49c4-866f-72cf0abc2417

frameshift MSH6 unknown 2fbc25da-3965-49c4-866f-72cf0abc2417

nonsense CDKN2A known 2fbc25da-3965-49c4-866f-72cf0abc2417

missense PRSS8 unknown 2fbc25da-3965-49c4-866f-72cf0abc2417

missense LRP1B unknown 2fbc25da-3965-49c4-866f-72cf0abc2417

missense IRF2 unknown cf11c31c-f4c3-48ba-9c46-66f406d0b7a1

missense IDH2 known cf11c31c-f4c3-48ba-9c46-66f406d0b7a1

missense DNMT3A unknown cf11c31c-f4c3-48ba-9c46-66f406d0b7a1

missense HSP90AA1 unknown cf11c31c-f4c3-48ba-9c46-66f406d0b7a1

missense RICTOR unknown 104ec531-5d95-41e2-ac72-f6cff2006b8e

nonframeshift ARID1B unknown 104ec531-5d95-41e2-ac72-f6cff2006b8e

frameshift TP53 likely 104ec531-5d95-41e2-ac72-f6cff2006b8e

missense TAF1 unknown 104ec531-5d95-41e2-ac72-f6cff2006b8e

nonsense TGFBR2 unknown 104ec531-5d95-41e2-ac72-f6cff2006b8e

missense MLL unknown 104ec531-5d95-41e2-ac72-f6cff2006b8e

missense KRAS known 104ec531-5d95-41e2-ac72-f6cff2006b8e

missense NSD1 unknown 104ec531-5d95-41e2-ac72-f6cff2006b8e

missense BRCA1 unknown 104ec531-5d95-41e2-ac72-f6cff2006b8e

missense DDR2 unknown 104ec531-5d95-41e2-ac72-f6cff2006b8e

missense TSC1 unknown 104ec531-5d95-41e2-ac72-f6cff2006b8e

missense FANCD2 unknown 104ec531-5d95-41e2-ac72-f6cff2006b8e

missense CCND2 unknown 104ec531-5d95-41e2-ac72-f6cff2006b8e

missense MAP3K1 unknown 104ec531-5d95-41e2-ac72-f6cff2006b8e

missense POLE unknown 104ec531-5d95-41e2-ac72-f6cff2006b8e

frameshift CYLD likely c7dbcfac-37ea-43f8-8899-1a9f2fb56341

missense BCORL1 unknown c7dbcfac-37ea-43f8-8899-1a9f2fb56341

missense SETD2 unknown c7dbcfac-37ea-43f8-8899-1a9f2fb56341

missense BLM unknown c7dbcfac-37ea-43f8-8899-1a9f2fb56341

missense FANCA unknown d199cfb0-91e8-471d-b1b3-53189cd64ee0

frameshift MSH6 unknown d199cfb0-91e8-471d-b1b3-53189cd64ee0

promoter TERT known d199cfb0-91e8-471d-b1b3-53189cd64ee0

missense MET unknown d199cfb0-91e8-471d-b1b3-53189cd64ee0

missense GPR124 unknown d199cfb0-91e8-471d-b1b3-53189cd64ee0

missense FGFR4 unknown d199cfb0-91e8-471d-b1b3-53189cd64ee0

missense TET2 unknown d199cfb0-91e8-471d-b1b3-53189cd64ee0

missense SOX10 unknown d199cfb0-91e8-471d-b1b3-53189cd64ee0

missense POLE unknown d199cfb0-91e8-471d-b1b3-53189cd64ee0

missense BRAF known d199cfb0-91e8-471d-b1b3-53189cd64ee0

missense RET unknown f0314175-2d19-4146-8754-fc5aed3ab420

nonframeshift DAXX unknown f0314175-2d19-4146-8754-fc5aed3ab420

missense JUN unknown f0314175-2d19-4146-8754-fc5aed3ab420

missense ARAF unknown f0314175-2d19-4146-8754-fc5aed3ab420

missense C11orf30 unknown f0314175-2d19-4146-8754-fc5aed3ab420

missense BCOR unknown f0314175-2d19-4146-8754-fc5aed3ab420

missense DAXX unknown f0314175-2d19-4146-8754-fc5aed3ab420

missense TET2 unknown f0314175-2d19-4146-8754-fc5aed3ab420

missense TSC2 unknown f0314175-2d19-4146-8754-fc5aed3ab420

missense JAK1 unknown f0314175-2d19-4146-8754-fc5aed3ab420

missense BRAF known f0314175-2d19-4146-8754-fc5aed3ab420

missense SPEN unknown f0314175-2d19-4146-8754-fc5aed3ab420

missense IDH1 known f0314175-2d19-4146-8754-fc5aed3ab420

missense SPEN unknown f0314175-2d19-4146-8754-fc5aed3ab420

missense PMS2 unknown 940171e7-d358-463a-8d9a-2b2fa90c2a84

missense CYLD unknown 940171e7-d358-463a-8d9a-2b2fa90c2a84

missense ERBB4 unknown 940171e7-d358-463a-8d9a-2b2fa90c2a84

nonsense TP53 known 940171e7-d358-463a-8d9a-2b2fa90c2a84

missense ZNF703 unknown 940171e7-d358-463a-8d9a-2b2fa90c2a84

nonsense MLL2 likely 940171e7-d358-463a-8d9a-2b2fa90c2a84

missense TSC2 unknown 940171e7-d358-463a-8d9a-2b2fa90c2a84

missense RUNX1 unknown 940171e7-d358-463a-8d9a-2b2fa90c2a84

missense CDH1 known 940171e7-d358-463a-8d9a-2b2fa90c2a84

missense FGFR1 unknown 940171e7-d358-463a-8d9a-2b2fa90c2a84

missense PREX2 unknown 131cf62d-ad78-49c1-a699-5bcc1004cd12

missense TP53 known 131cf62d-ad78-49c1-a699-5bcc1004cd12

nonframeshift WT1 unknown 131cf62d-ad78-49c1-a699-5bcc1004cd12

missense RAD50 unknown 131cf62d-ad78-49c1-a699-5bcc1004cd12

missense SLIT2 unknown 131cf62d-ad78-49c1-a699-5bcc1004cd12

missense FLCN unknown 131cf62d-ad78-49c1-a699-5bcc1004cd12

missense PDGFRA unknown 131cf62d-ad78-49c1-a699-5bcc1004cd12

missense KRAS known 131cf62d-ad78-49c1-a699-5bcc1004cd12

missense SPTA1 unknown 131cf62d-ad78-49c1-a699-5bcc1004cd12

missense MLL2 unknown 131cf62d-ad78-49c1-a699-5bcc1004cd12

missense MLL2 unknown 131cf62d-ad78-49c1-a699-5bcc1004cd12

Supplementary Table 2: Foundation One gene mutation frequency

gene N

KRAS 5

TP53 5

ARID1A 4

MLL2 4

TGFBR2 3

CDH1 3

FANCD2 3

PREX2 2

SPEN 2

APC 2

SETD2 2

FAT1 2

SLIT2 2

CTNNB1 2

DNMT3A 2

DAXX 2

LRP1B 2

GPR124 2

FANCA 2

ARID1B 2

MSH6 2

POLE 2

CYLD 2

TET2 2

BRAF 2

TSC2 2

CHEK1 1

EP300 1

DICER1 1

CREBBP 1

PDCD1LG2 1

STK11 1

ERBB2 1

FBXW7 1

KEAP1 1

CHEK2 1

RB1 1

CD79B 1

PRDM1 1

HNF1A 1

EMSY 1

RANBP2 1

FAM123B 1

VEGFA 1

ERBB3 1

NUP93 1

PIK3CA 1

MAGI2 1

PTCH1 1

SNCAIP 1

CTNNA1 1

KEL 1

CDK4 1

BRCA2 1

ALK 1

CSF1R 1

ATM 1

KIT 1

CDKN2A 1

PRSS8 1

IRF2 1

IDH2 1

HSP90AA1 1

RICTOR 1

TAF1 1

MLL 1

NSD1 1

BRCA1 1

DDR2 1

TSC1 1

CCND2 1

MAP3K1 1

BCORL1 1

BLM 1

TERT 1

MET 1

FGFR4 1

SOX10 1

RET 1

JUN 1

ARAF 1

C11orf30 1

BCOR 1

JAK1 1

IDH1 1

PMS2 1

ERBB4 1

ZNF703 1

RUNX1 1

FGFR1 1

WT1 1

RAD50 1

FLCN 1

PDGFRA 1

SPTA1 1

| **Days** | **Clinical** | **Genomic** | **Imaging** | **IPFS Hash** |
| --- | --- | --- | --- | --- |
| Multiple | ✔ | ✔ |  | [QmNecR9hX1eEjw2W9Bq7hKUPeinXvqfKKBJirX1Nxrmf4t](https://ipfs.infura.io/ipfs/QmNecR9hX1eEjw2W9Bq7hKUPeinXvqfKKBJirX1Nxrmf4t) |
| 26540 |  |  | ✔ | QmUcKAhkL4DwBTw5QM54eLzTmhtNoWzCBgwvWKh4XJeFwr |
| 26589 |  |  | ✔ | QmaYX3YvzDrendfcfnK1otff1kw88stxWM8XMUdsXXKSHP |
| 26645 |  |  | ✔ | QmQ6PtwhTMqw9b3SFsa1qfW79kGK7tPrhrUHpKVLtxmj1i |

Supplementary Table 3: All relevant CGT IPFS Hash information regarding patient c2e2e081-4c39-4201-8a27-7b469ed39490

Supplementary Table 4: Registry scores for each data element per patient

| Pt # | E1 | E2 | E3 | E4 | E5 | E6 | E7 | E8 | E9 | E10 |
| --- | --- | --- | --- | --- | --- | --- | --- | --- | --- | --- |
| f9b6a782-bbf5-4be8-bf7e-d1a9586d9552 | 5 | 1 | 1 | 5 | 5 | 5 | 5 | 4 | 5 | 3 |
| c2e2e081-4c39-4201-8a27-7b469ed39490 | 5 | 5 | 5 | 5 | 3 | 5 | 5 | 5 | 3 | 0 |
| db2d85aa-4f94-4e77-8755-6b94a710c1aa | 5 | 5 | 5 | 5 | 5 | 4 | 5 | 5 | 3 | 0 |
| 2fbc25da-3965-49c4-866f-72cf0abc2417 | 5 | 5 | 5 | 5 | 5 | 5 | 5 | 5 | 5 | 3 |
| 940171e7-d358-463a-8d9a-2b2fa90c2a84 | 5 | 5 | 1 | 5 | 5 | 0 | 5 | 5 | 0 | 0 |
| f0314175-2d19-4146-8754-fc5aed3ab420 | 5 | 5 | 1 | 5 | 3 | 0 | 5 | 5 | 0 | 0 |
| c7dbcfac-37ea-43f8-8899-1a9f2fb56341 | 0 | 0 | 0 | 0 | 5 | 0 | 5 | 5 | 0 | 0 |
| ef5c3164-6f45-4d3a-88f0-4509226c5571 | 5 | 5 | 5 | 5 | 5 | 5 | 5 | 5 | 5 | 5 |
| ec3d977b-c310-4df3-a444-f79bc3dd8b58 | 5 | 5 | 5 | 5 | 5 | 0 | 5 | 5 | 0 | 0 |
| 131cf62d-ad78-49c1-a699-5bcc1004cd12 | 5 | 5 | 5 | 5 | 5 | 0 | 5 | 5 | 0 | 0 |
| cf11c31c-f4c3-48ba-9c46-66f406d0b7a1 | 5 | 5 | 5 | 5 | 5 | 5 | 4 | 5 | 4 | 4 |
| ccc2ba97-912f-4b62-b767-cca129ee6a56 | 0 | 0 | 0 | 0 | 3 | 2 | 5 | 3 | 0 | 0 |
| 104ec531-5d95-41e2-ac72-f6cff2006b8e | 5 | 5 | 5 | 5 | 5 | 0 | 5 | 5 | 0 | 0 |
| a5627ac3-450d-4036-ade8-99ae62a5c232 | 5 | 5 | 5 | 5 | 5 | 5 | 5 | 5 | 5 | 0 |
| 5189efbe-3382-4353-ad2f-9afd0255c2c8 | 5 | 5 | 5 | 5 | 5 | 5 | 5 | 5 | 4 | 3 |
| 253f0e2d-bebd-464b-81c5-8dd8385192b3 | 5 | 5 | 5 | 5 | 3 | 5 | 5 | 5 | 5 | 3 |
| d199cfb0-91e8-471d-b1b3-53189cd64ee0 | 5 | 5 | 5 | 5 | 3 | 5 | 5 | 3 | 5 | 3 |

Per patient breakdown of registry scores for each data element. The data elements are numbered accordingly: E1 is gender; E2 is ethnicity; E3 is race; E4 is year of birth; E5 is date of diagnosis; E6 is basis of diagnosis; E7 is cancer site; E8 is cancer histology; E9 is therapeutic agent/modality; E10 is beginning and end dates of treatment.

Supplementary Table 5: OMOP scores for each data element per patient

| Pt # | E1 | E2 | E3 | E4 | E5 | E6 | E7 | E8 | E9 | E10 |
| --- | --- | --- | --- | --- | --- | --- | --- | --- | --- | --- |
| f9b6a782-bbf5-4be8-bf7e-d1a9586d9552 | 5 | 5 | 1 | 5 | 1 | 3 | 4 | 0 | 2 | 2 |
| c2e2e081-4c39-4201-8a27-7b469ed39490 | 5 | 5 | 5 | 5 | 0 | 2 | 5 | 3 | 2 | 2 |
| db2d85aa-4f94-4e77-8755-6b94a710c1aa | 5 | 5 | 5 | 5 | 0 | 3 | 5 | 0 | 2 | 2 |
| 2fbc25da-3965-49c4-866f-72cf0abc2417 | 5 | 5 | 5 | 5 | 0 | 5 | 5 | 0 | 0 | 0 |
| 940171e7-d358-463a-8d9a-2b2fa90c2a84 | 5 | 5 | 5 | 5 | 5 | 5 | 5 | 0 | 3 | 3 |
| f0314175-2d19-4146-8754-fc5aed3ab420 | 5 | 5 | 5 | 5 | 3 | 5 | 5 | 0 | 3 | 3 |
| c7dbcfac-37ea-43f8-8899-1a9f2fb56341 | 5 | 5 | 5 | 5 | 5 | 3 | 5 | 0 | 0 | 0 |
| ef5c3164-6f45-4d3a-88f0-4509226c5571 | 5 | 5 | 5 | 5 | 3 | 2 | 4 | 0 | 0 | 0 |
| ec3d977b-c310-4df3-a444-f79bc3dd8b58 | 5 | 5 | 5 | 5 | 5 | 2 | 4 | 0 | 2 | 0 |
| 131cf62d-ad78-49c1-a699-5bcc1004cd12 | 5 | 5 | 5 | 5 | 3 | 5 | 5 | 0 | 0 | 0 |
| cf11c31c-f4c3-48ba-9c46-66f406d0b7a1 | 5 | 5 | 5 | 5 | 3 | 0 | 2 | 4 | 0 | 0 |
| ccc2ba97-912f-4b62-b767-cca129ee6a56 | 5 | 5 | 5 | 5 | 3 | 2 | 5 | 3 | 0 | 0 |
| 104ec531-5d95-41e2-ac72-f6cff2006b8e | 5 | 5 | 1 | 5 | 3 | 0 | 5 | 0 | 0 | 0 |
| a5627ac3-450d-4036-ade8-99ae62a5c232 | 5 | 5 | 5 | 5 | 3 | 3 | 5 | 0 | 3 | 0 |
| 5189efbe-3382-4353-ad2f-9afd0255c2c8 | 5 | 5 | 5 | 5 | 5 | 3 | 5 | 0 | 3 | 2 |
| 253f0e2d-bebd-464b-81c5-8dd8385192b3 | 5 | 5 | 5 | 5 | 2 | 0 | 5 | 5 | 5 | 0 |
| d199cfb0-91e8-471d-b1b3-53189cd64ee0 | 5 | 5 | 5 | 5 | 2 | 0 | 5 | 0 | 3 | 3 |

Per patient breakdown of OMOP scores for each data element. The data elements are numbered accordingly: E1 is gender; E2 is ethnicity; E3 is race; E4 is year of birth; E5 is date of diagnosis; E6 is basis of diagnosis; E7 is cancer site; E8 is cancer histology; E9 is therapeutic agent/modality; E10 is beginning and end dates of treatment.

**Supplementary References**

1. Emam KE, Dankar FK, Vaillancourt R, Roffey T, Lysyk M. Evaluating the Risk of Re-identification of Patients from Hospital Prescription Records. Can J Hosp Pharm. 2009 Jul;62(4):307-19. PMID: 22478909.

2. , , , , , , et al.; Available from: https://www.hhs.gov/hipaa/for-professionals/privacy/special-topics/de-identification/index.html.

3. Conley RB, Dickson D, Zenklusen JC, Al Naber J, Messner DA, Atasoy A, et al. Core Clinical Data Elements for Cancer Genomic Repositories: A Multi-stakeholder Consensus. Cell. 2017 Nov 16;171(5):982-6. PMID: 29149611. doi: 10.1016/j.cell.2017.10.032.
